# Acute superior mesenteric artery syndrome in a young adult: case report

**DOI:** 10.1097/MS9.0000000000005693

**Published:** 2026-09-16

**Authors:** Mohamad Khalife, Mohamad E. Haress, Riwa Deghaim, Mustapha El Lakis, Hadeel Ghaddar, Mustafa Saad El Masri

**Affiliations:** Department of General Surgery, American University of Beirut Medical Center, Beirut, Lebanon

**Keywords:** aortomesenteric compression, case report, duodenal obstruction, minimally invasive surgery, rapid weight loss, Wilkie syndrome

## Abstract

**Introduction and importance:**

Superior mesenteric artery (SMA) syndrome is a rare condition (incidence of 0.013%–0.78%) that primarily affects young adults, resulting from duodenal compression between the SMA and the aorta following rapid weight loss and retroperitoneal fat pad depletion. Conservative management succeeds in 70–80% of cases; surgical intervention is required for refractory symptoms.

**Case presentation:**

A 21-year-old woman presented with severe abdominal pain, nausea, and bilious vomiting following a 36% weight reduction over 5 months (66–42 kg; body mass index [BMI] 17.48 kg/m^2^). CT revealed gastroduodenal dilatation with an aortomesenteric angle of 18° (normal 28–65°) and a distance of 4.5 mm (normal 10–34 mm), confirming SMA syndrome. After 4 days of unsuccessful conservative management, laparoscopic surgery was initiated with Strong’s procedure. Periduodenal fibrosis caused persistent obstruction, requiring immediate conversion to extracorporeal duodenojejunostomy. Recovery was uncomplicated, with oral intake by postoperative day 6. At the 2-month follow-up, she achieved complete symptom resolution with an 8 kg weight gain (BMI 20.81 kg/m^2^).

**Clinical discussion:**

When Strong’s procedure fails due to periduodenal fibrosis, immediate intraoperative conversion to duodenojejunostomy is safe and effective, sparing a malnourished patient a second procedure. Review of 20 surgically managed cases (2011–2025) confirms that most proceed directly to duodenojejunostomy; this fibrosis-driven conversion strategy is infrequently described and offers a practical framework for intraoperative decision-making in high-risk patients.

**Conclusion:**

Early surgical consultation is recommended when conservative treatment fails. In malnourished patients with suspected periduodenal fibrosis, preoperative planning should anticipate possible intraoperative conversion; immediate bypass during the same operation avoids a second procedure and its associated morbidity.

## Introduction

Superior mesenteric artery (SMA) syndrome, also known as Wilkie syndrome, is a rare condition with an estimated incidence of 0.013–0.78% in the general population^[^1^]^. However, the true prevalence remains unknown due to variable diagnostic criteria and potential under- or over-diagnosis in clinical practice^[^2^]^. Approximately 75% of cases occur in patients aged 10–30 years^[^3^]^, with the pediatric population most commonly affected secondary to acute weight loss or following spinal surgery^[^4^]^.

HIGHLIGHTS

- Rapid weight loss causing aortomesenteric fat pad depletion can precipitate acute superior mesenteric artery syndrome; computed tomography measurements (angle ≤18°, distance ≤4.5 mm) remain the diagnostic cornerstone.
- Strong’s procedure alone may fail in the presence of chronic periduodenal fibrosis, and immediate intraoperative conversion to duodenojejunostomy avoids a second procedure in an already malnourished patient.
- When conservative management fails, early surgical intervention, with a flexible, stepwise approach, facilitates complete symptom resolution and nutritional recovery.

The syndrome results from compression of the third portion of the duodenum between the SMA and the abdominal aorta, typically due to loss of the retroperitoneal fat pad^[^2^]^. Normally, the duodenum passes through the aortomesenteric space with adequate clearance maintained by retroperitoneal and mesenteric adipose tissue. When this fat cushion is depleted through rapid weight loss, the aortomesenteric angle narrows, and the distance between these vessels decreases, leading to duodenal compression and obstruction.

Computed tomography (CT) with aortomesenteric parameter measurement is the diagnostic gold standard, with normal aortomesenteric angles ranging from 28° to 65° and distances from 10 to 34 mm^[^5^]^. Treatment approaches range from conservative nutritional support and postural therapy to surgical intervention, with duodenojejunostomy being the most effective surgical option when conservative management fails. While conservative management succeeds in 70–80% of cases, surgical intervention may be required for refractory symptoms^[^2^]^.

This case report presents a young woman with SMA syndrome who was managed with laparoscopic surgery and provides context from 20 surgically managed cases documented between 2011 and 2025 (Supplemental Digital Content Tables S1 and S2, available at: https://links.lww.com/MS9/B388). The primary aim of this report is to document the intraoperative decision-making process when Strong’s procedure fails due to periduodenal fibrosis and to demonstrate that immediate conversion to duodenojejunostomy within the same operation is a safe, effective strategy that spares a malnourished patient a second procedure – a fibrosis-driven conversion scenario that is rarely described in detail in the literature. This report is particularly relevant given the increasing prevalence of rapid weight-loss practices among young adults. This case report is reported in line with the SCARE 2025 Checklist^[^6^]^.

## Case presentation

### Chief complaints

A previously healthy 21-year-old woman presented to the emergency department with a 2-day history of severe, cramping abdominal pain of increasing intensity, associated with nausea and multiple episodes of bilious, foul-smelling emesis.

### History of present illness

The patient reported severe cramping abdominal pain that was non-radiating and had been progressively worsening over 2 days. She was unable to tolerate any oral intake and had not passed a bowel movement for the past 10 days, though she had passed flatus the day before presentation. The delayed medical presentation likely reflects a combination of factors, including the patient’s young age, gradual symptom progression, and prior episodes of self-limiting postprandial discomfort that she attributed to her dietary restriction. CT on admission showed no mechanical colonic obstruction, suggesting that the prolonged constipation represented progressive duodenal dysmotility rather than large-bowel pathology. She denied fever or other systemic symptoms.

### History of past illness

The patient had no significant past medical or surgical history and was not taking any medications. The patient had intentionally lost 24 kg over 5 months (66–42 kg; body mass index [BMI], 27.5–17.48 kg/m^2^) through strict dieting and exercise. Her dietary intake became severely limited to approximately one cup of yogurt daily, with progressive postprandial fullness and discomfort.

### Physical examination

The patient appeared thin and distressed but alert and oriented. Vital signs were stable. Physical measurements: height, 155 cm; weight, 42 kg; BMI, 17.48 kg/m^2^. Abdominal examination revealed distension with diffuse tenderness on palpation.

### Laboratory examinations

Laboratory investigations revealed mild hypernatremia (147 mEq/L) and a slightly elevated C-reactive protein level (4.3 mg/L). The complete blood count, renal function, and liver enzymes were within normal limits.

### Imaging examinations

CT of the abdomen and pelvis with intravenous contrast demonstrated marked dilatation of the stomach and the first and second duodenal segments, with a transition point at the aortomesenteric region. The third portion of the duodenum showed significant caliber reduction and appeared compressed between the SMA and the abdominal aorta. The aortomesenteric angle measured 18° (normal 28°–65°), and the aortomesenteric distance was 4.5 mm (normal 10–34 mm). Sagittal reconstruction clearly demonstrated the narrowed aortomesenteric space with duodenal compression (Fig. 1). These findings were diagnostic of SMA syndrome.

**Figure 1.**
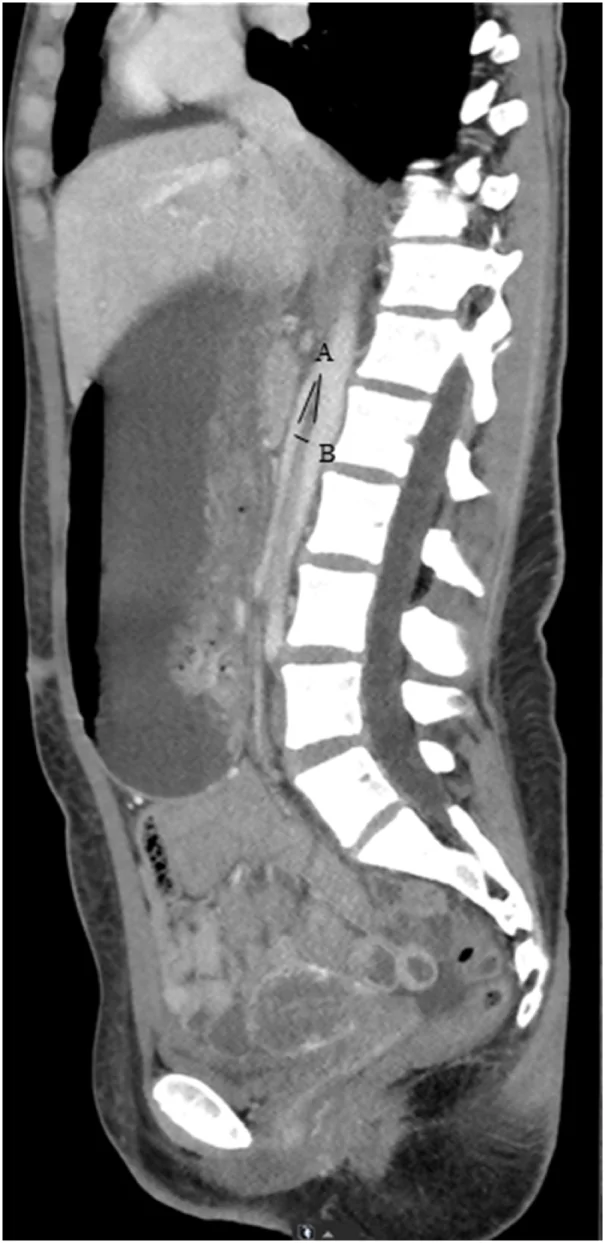
Sagittal view of abdominal computed tomography showing the superior mesenteric artery–aorta angle (A = 18°) and the aortomesenteric distance (B = 4.5 mm).

SMA syndrome is secondary to rapid weight loss, with pathological narrowing of the aortomesenteric space causing duodenal compression.

### Treatment

Conservative management with nasogastric decompression, intravenous fluids, and total parenteral nutrition (with thiamine supplementation) was initiated. Nasojejunal tube placement was considered but not pursued, given the severity of duodenal obstruction on imaging and the patient’s clinical trajectory. After 4 days without significant improvement, and in the context of clinical deterioration with persistent complete obstruction, ongoing bilious nasogastric drainage, and inability to tolerate any oral or enteral intake, surgical intervention was pursued. Electrolyte optimization was performed alongside nutritional support throughout the conservative phase.

The patient was placed in the supine position. The skin was prepared with chlorhexidine and draped in a sterile fashion. Under general anesthesia, after administration of prophylactic ceftizoxime (Cefizox) 1 g, a supraumbilical 10-mm Hasson port and three 5-mm ports were inserted. Exploration revealed gastric and proximal duodenal dilation. The ligament of Treitz was identified and divided (Strong’s procedure) to relieve duodenal compression. Despite adequate mobilization, intraoperative findings of periduodenal fibrotic tissue and adhesions, consistent with chronic inflammation, caused persistent obstruction, prompting conversion to an extracorporeal duodenojejunostomy.

A 5 cm upper midline incision allowed exteriorization of the duodenum and of a jejunal loop approximately 30 cm distal to the ligament of Treitz. A side-to-side functional end-to-end duodenojejunostomy was created using 4-0 and 3-0 PDS sutures. The anastomosis was returned to the peritoneal cavity, and the abdomen was closed in standard fashion. No surgical drains were placed.

### Outcome and follow-up

The patient had an uncomplicated recovery. On postoperative day 5, Gastrografin swallow confirmed free contrast flow through the duodenojejunostomy without leak. Subsequent imaging demonstrated patent anastomosis and complete resolution of duodenal compression (Figs. 2–4). The patient tolerated oral intake by postoperative day 6 and was discharged in good condition.

**Figure 2.**
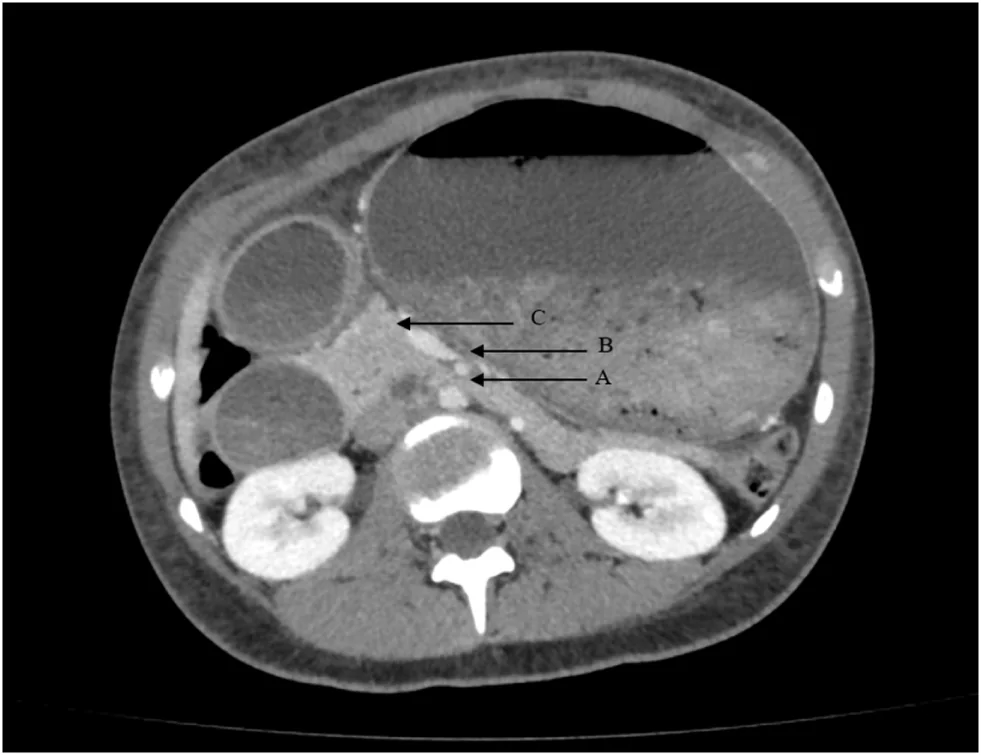
Axial view of abdominal computed tomography showing duodenal compression between the aorta (A) and the superior mesenteric artery (B), with distended bowel (C).

**Figure 3.**
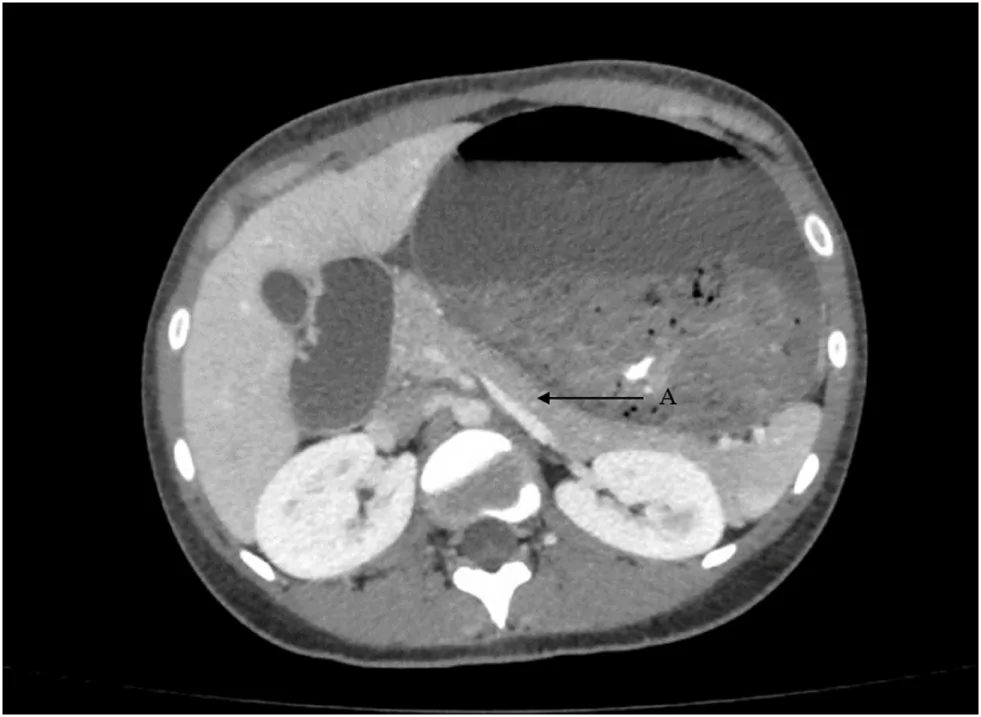
Postoperative axial abdominal computed tomography view demonstrating resolution of duodenal compression with a non-distended small bowel. The patent left renal vein (A) is an incidental radiological finding without clinical significance for the diagnosis or management of SMA syndrome.

**Figure 4.**
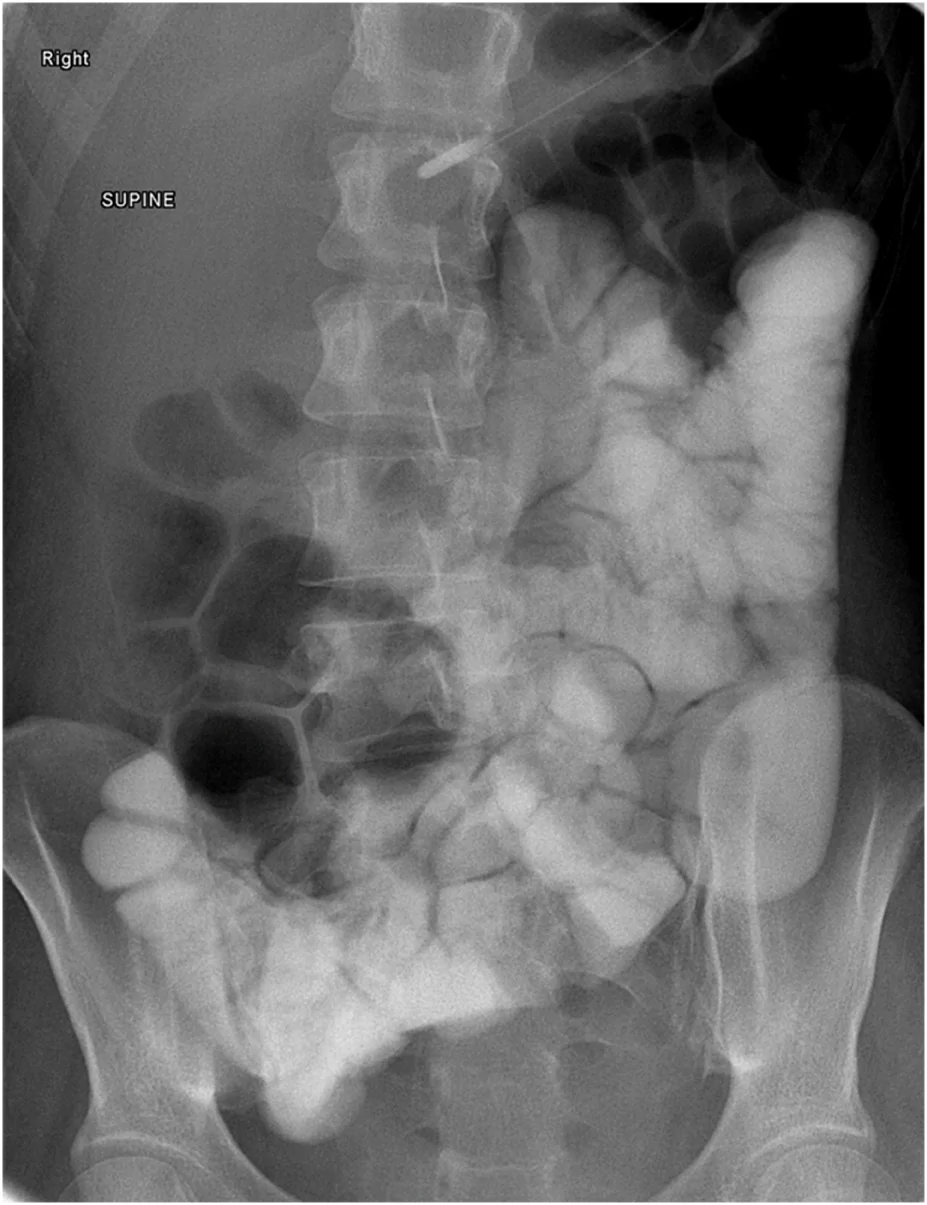
Postoperative Gastrografin swallow showing a patent duodenojejunostomy anastomosis with free passage into the small bowel.

### Final diagnosis

At 2-month follow-up, the patient had gained 8 kg (BMI 20.81 kg/m^2^), was tolerating a regular diet, and reported complete resolution of obstructive symptoms. Throughout the postoperative period, the patient was followed by a clinical dietitian with structured dietary advancement from clear liquids to a soft diet, guided by nutritional targets set collaboratively with the surgical and nutrition teams.

## Discussion

While conservative management succeeds in approximately 70–80% of cases^[^2^]^, surgical intervention becomes necessary when initial therapy fails, with a reported recurrence rate of 15.8%^[^7^]^. To contextualize this case, a literature search was conducted using PubMed/MEDLINE and Embase, with the search terms “superior mesenteric artery syndrome,” “Wilkie syndrome,” “duodenojejunostomy,” and “Strong’s procedure,” limited to English-language publications from January 2011 to March 2025. Inclusion criteria comprised adult or adolescent patients managed surgically, with documented operative technique and outcome data, yielding 20 cases (Supplemental Digital Content Tables S1 and S2, available at: https://links.lww.com/MS9/B388). As is standard for case-report contextual reviews, a narrative approach was employed rather than a formal systematic review, given the descriptive and clinical, rather than inferential, aim of this synthesis.

While intraoperative conversion from Strong’s procedure to duodenojejunostomy has been described in the literature, prior reports have described this as a response to unexpected intraoperative complications such as bowel ischemia or dense adhesions^[^8,9^]^. This case illustrates real-time intraoperative reassessment when Strong’s procedure proved insufficient due to periduodenal fibrosis – a scenario seldom detailed in prior reports. Rather than closing and deferring definitive repair, the surgical team proceeded immediately to extracorporeal duodenojejunostomy within the same operation, avoiding a second procedure and demonstrating the value of intraoperative flexibility in malnourished patients, where the extent of duodenal scarring may be uncertain preoperatively. This approach spared the patient a second general anesthetic and operation, which carries added risk in a severely malnourished young adult.

Laparoscopic duodenojejunostomy has emerged as the preferred surgical approach, demonstrating success rates of 80–100% with minimal complications^[^2^]^. Among the 20 surgically managed cases identified, the median age was 20 years, with a female predominance and a median BMI of 17.3 kg/m^2^, consistent with our patient’s presentation. Radiologic parameters showed a median aortomesenteric angle of 14° (range 7–25°) and an aortomesenteric distance of 4.5 mm (range 3.6–7.8 mm), comparable to our patient’s measurements. Long-term data from published series are limited, but reported recurrence rates after duodenojejunostomy range from 0 to 15.8%, with most patients maintaining weight gain and symptom resolution at 1–2 years of follow-up^[^7,10^]^.

The decision to proceed with surgery after 4 days of conservative management warrants discussion. The recommendation of 4–6 weeks of conservative therapy applies to hemodynamically stable patients who can tolerate at least partial enteral or parenteral nutrition and show signs of clinical improvement^[^11^]^. Our patient met none of these criteria: she had clinically deteriorated despite 4 days of adequate IV nutrition and electrolyte correction, had persistent complete duodenal obstruction with ongoing high-volume bilious nasogastric drainage, and could not tolerate any oral or enteral intake. Nasojejunal feeding, the preferred bridge in stable patients, was not feasible given the severity of obstruction on imaging. It is well established that no formal consensus exists on the optimal duration of conservative management^[^12^]^, that reported durations before surgery range from 2 days to over 5 months^[^13^]^, and that surgery is indicated when conservative measures fail and enteral access is not achievable^[^4,14^]^. In this context, proceeding at 4 days was not premature but clinically appropriate. Early surgical intervention in such cases has been observed to prevent complications of prolonged gastric obstruction, including irreversible impairment of gastric motor function^[^10^]^.

We employed a stepwise intraoperative strategy, beginning with laparoscopic Strong’s procedure (division of the ligament of Treitz). This initial step was chosen given the patient’s severe malnutrition (BMI 17.48 kg/m^2^), in whom avoiding intestinal anastomosis could theoretically reduce perioperative risk. The laparoscopic approach allowed real-time intraoperative assessment of duodenal mobility following mobilization. When persistent duodenal obstruction was identified, due to chronic compression and scarring, we proceeded intraoperatively to an extracorporeal duodenojejunostomy, achieving definitive treatment.

This approach differs from proceeding directly to duodenojejunostomy, which many surgeons appropriately employ as first-line management given its established high success rates. Strong’s procedure alone has a reported failure rate of up to 25%^[^11^]^ and can be limited by anatomical factors, such as interference from short vessels of the inferior pancreaticoduodenal artery^[^8^]^. This case illustrates the intraoperative decision-making process when initial mobilization proves inadequate, highlighting the importance of surgical flexibility and readiness to proceed with definitive bypass.

A literature analysis revealed that 60% of reviewed cases utilized laparoscopic techniques, demonstrating excellent outcomes with reduced pain, faster recovery, and superior cosmesis^[^2,15^]^. The laparoscopic approach with extracorporeal anastomosis combines minimally invasive benefits with direct visualization for the critical anastomosis. The ability to convert to open approaches, when indicated, ensures optimal outcomes^[^9^]^.

While both gastrojejunostomy and duodenojejunostomy can bypass the obstruction, duodenojejunostomy is preferred as it addresses the duodenal obstruction directly. Gastrojejunostomy bypasses the stomach rather than the duodenum and is associated with long-term complications, including bile reflux gastritis, marginal ulceration, and blind loop syndrome^[^16^]^. Surgical intervention provides definitive anatomical correction, whereas conservative management, though successful in 70–80% of cases, does not address the fundamental mechanical compression and may result in symptom recurrence in some patients^[^11^]^.

The patient’s severe dietary restriction and rapid weight loss warrant consideration of an underlying eating disorder pathology. However, psychiatric evaluation revealed no formal eating disorder diagnosis in this case. Her successful postoperative weight gain from 42 to 50 kg (a 19% increase), bringing her BMI from WHO Grade I thinness (17.48 kg/m^2^)^[^13^]^ to the normal range (20.81 kg/m^2^), demonstrates effective nutritional recovery. In cases where eating disorders are identified, multidisciplinary management, including psychiatric support and long-term follow-up, is essential to monitor weight maintenance and prevent recurrence^[^17^]^.

Our patient achieved complete resolution of symptoms with sustained weight gain at the 2-month follow-up, demonstrating excellent short-term surgical outcomes.

### Limitations

As with all case reports, the findings presented here are descriptive and hypothesis-generating rather than definitive. The value of this report lies in its detailed documentation of an intraoperative decision-making process that is infrequently described in the literature. The 2-month follow-up captured complete resolution of obstructive symptoms and significant weight restoration, representing the primary surgical endpoints; longer-term surveillance would further characterize the durability of these outcomes, particularly given that published recurrence rates after duodenojejunostomy range up to 15.8%^[^7^]^. The periduodenal fibrotic changes identified intraoperatively were assessed macroscopically, which is consistent with standard surgical practice in this setting; histopathological sampling was not performed as it would not have altered the operative decision or patient management. The literature search employed a narrative approach appropriate for case report contextual reviews. Psychiatric evaluation during admission did not identify a formal eating disorder diagnosis; the patient’s dietary behavior was addressed through nutritional counseling, and psychological follow-up was discussed with the patient and family.

## Conclusion

This case demonstrates that when Strong’s procedure fails intraoperatively due to periduodenal fibrosis, immediate conversion to duodenojejunostomy within the same operation is safe, effective, and avoids the added morbidity of a second procedure in an already malnourished patient. This fibrosis-driven conversion scenario is infrequently described in the literature; recognizing it preoperatively and planning for it represent the key practical lesson from this case. Early recognition through CT imaging, prompt surgical consultation, and multidisciplinary nutritional support remain essential for optimal outcomes.

## Data Availability

All data relevant to this case are included in the article. Additional information may be obtained from the corresponding author upon reasonable request.

## Research registration unique identifying number (UIN)

Not applicable. This is a case report and was not registered as a clinical study.
